# Evaluation of Deterioration Parameters of Hazelnut Oil, Palm Olein and Their Blends During Repeated Deep‐Frying of Potatoes

**DOI:** 10.1002/fsn3.71726

**Published:** 2026-04-03

**Authors:** Ayhan Baştürk, M. Murat Ceylan

**Affiliations:** ^1^ Department of Food Engineering, Faculty of Engineering Van Yuzuncu Yil University Van Turkey; ^2^ School of Tourism and Hotel Management, Department of Gastronomy and Culinary Arts Hatay Mustafa Kemal University Hatay Turkey

**Keywords:** 3‐MCPD ester, blended oils, deep‐frying, glycidyl ester, hazelnut oil, oxidative stability, palm olein, total polar compounds

## Abstract

This study investigated the deterioration behavior of hazelnut oil (HO), palm olein (PO), their blends (B‐1: 34% PO + 66% HO; B‐2: 66% PO + 34% HO), and shortening (S) during 12 consecutive deep‐frying cycles of potato slices at 180°C. Changes in free fatty acids (FFA), peroxide value (PV), p‐anisidine value (p‐AV), total oxidation value (TOTOX), conjugated dienes (K_232_) and trienes (K_270_), color (L*, a*, b*), viscosity, total polar compounds (TPC), fatty acid profile, and heat‐induced contaminants 3‐monochloropropane‐1,2‐diol esters (3‐MCPD) and glycidyl esters (GE) were monitored to assess oxidative and physicochemical stability. HO, despite its high oleic acid content, exhibited the fastest deterioration, showing substantial increases in PV, p‐AV, TOTOX, and spectrophotometric indices. PO demonstrated superior thermal stability due to its higher palmitic and oleic acid levels, while S showed the lowest overall deterioration but accumulated the highest levels of 3‐MCPD and GE. Blends exhibited intermediate performance, with B‐2 outperforming B‐1, indicating the positive contribution of PO to oxidative resistance. TPC values in all samples remained below the regulatory limit of 25%, although progressive viscosity increases reflected polymerization during frying. The formation of 3‐MCPD and GE intensified with frying cycles, particularly in PO and S, underscoring the importance of contaminant control in high‐temperature applications. Overall, blending HO with PO improved oxidative stability and frying performance while maintaining acceptable quality indices. These findings support the potential of optimized HO—PO blends as economically and technologically viable frying oils.

## Graphical abstract

This study systematically investigates the evolution of oxidative degradation and the formation of process contaminants (3‐MCPD and glycidyl esters) in hazelnut oil, palm olein, their blends, and shortening during repeated frying cycles. Multiple analytical parameters, including FFA, PV, p‐anisidine value, conjugated dienes/trienes, and Totox, were monitored. Hazelnut oil showed the most rapid degradation, whereas shortening exhibited the lowest oxidation levels but the highest 3‐MCPD/GE accumulation. The results demonstrate that improved oxidative stability does not necessarily correlate with reduced contaminant formation, emphasizing the need for balanced oil formulation strategies in frying systems.

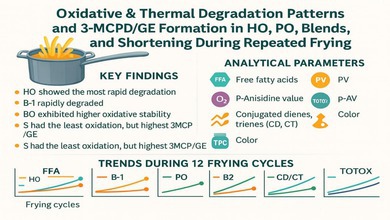

## 1 Introduction

Deep‐frying is one of the most common thermal processing techniques used worldwide in both household cooking and industrial food manufacturing. The global demand for fried foods has steadily increased, driving a parallel rise in frying‐oil consumption and emphasizing the economic importance of maintaining oil quality during repeated use (FAO 2025). Because frying oils constitute a significant portion of the edible‐oil market, understanding their degradation behavior is essential for ensuring both safety and product quality. During deep frying, oils are exposed to high temperatures (160°C–190°C) in the presence of air and moisture, initiating oxidation, hydrolysis, and polymerization reactions (López et al. 2024). These processes generate free fatty acids (FFA), peroxides, aldehydes, and high‐molecular‐weight polymers, all of which adversely affect sensory attributes and nutritional value (Silva et al. 2024; Tan et al. 2025). Additionally, heat‐induced processing contaminants such as 3‐monochloropropane‐1,2‐diol esters (3‐MCPD) and glycidyl esters (GE) may form, posing toxicological risks (EFSA 2016; Shi et al. 2023). International frameworks, including the Codex Alimentarius (CXS 210–1999) and EU Regulation 2022/2104, establish strict limits for both oxidative deterioration and contaminant formation. Oils are typically discarded once total polar compounds (TPC) exceed ~25%, while regulatory limits for 3‐MCPD and GE remain around 2.5 and 1.0 mg/kg, respectively (EU 2020). These legislative benchmarks emphasize the importance of continuous oil‐quality monitoring using both classical oxidation indices and contaminant markers. The stability of frying oils depends on several intrinsic and extrinsic factors. Fatty‐acid composition, especially the ratio of saturated to unsaturated fatty acids, strongly influences oxidative behavior. Although natural antioxidants such as tocopherols delay oxidation, they are gradually depleted during heating. In recent years, increasing attention has been given to the use of natural antioxidant compounds to improve the oxidative stability of frying oils and to protect food quality during high‐temperature processing, although their effectiveness may depend on the type of process, food matrix, and antioxidant composition (Juncos et al. 2025). Frying temperature, duration, moisture content, and food‐to‐oil ratio also affect degradation kinetics, while metal ions and food residues can catalyze polymerization reactions. Therefore, comprehensive assessment of frying stability requires integrating multiple chemical and physical quality indicators. Oil degradation is routinely monitored through indices such as FFA, peroxide value (PV), p‐anisidine value (p‐AV), and total oxidation (TOTOX), along with UV spectrophotometric markers (K_232_, K_270_) that reflect conjugated diene and triene formation (Ceylan and Baştürk 2023; Shahidi and Zhong 2005). Physical parameters including color and viscosity provide additional insight, as polymerization increases viscosity and pigment formation causes darkening (Sadawarte and Annapure 2023). Among these markers, TPC remains the most robust global indicator for determining the endpoint of frying oil (Codex Alimentarius 1999; Weisshaar 2014). Hazelnut oil (HO), rich in oleic acid and α‐tocopherol, is nutritionally valuable but displays limited oxidative stability during prolonged heating (Cakmak‐Arslan and Gulsen 2024). In contrast, palm olein (PO), with its balanced palmitic‐oleic profile, exhibits superior thermal resistance and is widely used in commercial frying operations (Akinoso et al. 2025). Blending HO with PO offers the potential to combine their respective nutritional and functional advantages, yet studies on the frying behavior of HO‐PO blends particularly concerning contaminant formation remain scarce.

Although numerous studies have investigated the frying stability of individual edible oils, research on the frying performance of oil blends remains relatively limited. Blending different vegetable oils is increasingly considered a practical approach to balance nutritional quality, oxidative stability, and economic cost in industrial frying applications. However, most previous studies on oil blends have primarily focused on conventional oxidation indicators such as peroxide value, free fatty acids, or total polar compounds. Comparatively little attention has been given to the formation of heat‐induced processing contaminants, particularly 3‐monochloropropane‐1,2‐diol esters (3‐MCPD) and glycidyl esters (GE), in blended frying oils during repeated frying cycles. Considering the widespread industrial use of palm olein and the nutritional importance of hazelnut oil, a comprehensive evaluation of their blends under realistic frying conditions is needed to better understand both quality deterioration and contaminant formation.

The novelty of this study lies in the simultaneous evaluation of oxidative degradation, physicochemical changes, and heat‐induced contaminant formation in hazelnut oil–palm olein blends during repeated deep‐frying cycles. Understanding the behavior of such blends is particularly relevant for the food industry, where blending different vegetable oils is commonly used to optimize frying stability, nutritional quality, and economic feasibility.

This study aimed to comprehensively evaluate the changes in physicochemical properties and heat‐induced contaminants in HO, PO, their blends B‐1 (34% PO + 66% HO) and B‐2 (66% PO + 34% HO), and shortening (S) over 12 consecutive frying cycles. Quality parameters including FFA, PV, p‐AV, TOTOX, K_232_/K_270_, color, viscosity, TPC, fatty‐acid composition, and 3‐MCPD + GE were monitored to characterize the relationship between oxidative degradation, physical changes, and frying performance within the context of international food‐quality standards.

## 2 Materials and Methods

### 2.1 Materials

Palm olein (PO) used in this study was imported from Malaysia and supplied by Orkide Oils Inc. (Izmir, Turkey). The oil had undergone double refining in 2017. Refined hazelnut oil (HO), obtained from the Turkish Tombul hazelnut variety, was procured from Fiskobirlik Inc. (Ordu, Turkey). The blend ratios (34% and 66%) were chosen to obtain two formulations with contrasting dominance of hazelnut oil or palm olein, enabling evaluation of the influence of oil composition on frying stability and contaminant formation. Commercial shortening (S), produced by Besler Inc. (Bursa, Turkey), was purchased from a local market. The shortening consisted of a vegetable‐based, anhydrous frying fat containing varying proportions of palm‐derived oils and was included as a control sample for comparative purposes. During the frying trials, Agria‐variety potatoes (Nevşehir, Turkey) from a single batch were used to ensure uniformity. Their composition was as follows: dry matter 21.4% ± 0.3%, reducing sugars 0.12% ± 0.01%, and starch 17.9% ± 0.5%. Chemicals including 3‐MCPD, 3‐chloro‐1,2‐propane‐1,1,2,3,3‐d5‐diol (3‐MCPD‐d5), glycidyl stearate, diethyl ether, methanol, sodium hydroxide, sodium bromide, ethyl acetate, phenylboronic acid (PBA), acetone, and toluene were purchased from Sigma‐Aldrich (Steinheim, Germany). All reagents were of analytical grade.

### 2.2 Sample Preparation

Potatoes were peeled, washed, and dried using paper towels, then cut into uniform 1 × 1 × 3 cm slices using a mechanical slicer (Metaltex Fritex, Switzerland). To minimize oil absorption and enzymatic browning, the slices were soaked in a 2.5% brine solution for 5 min prior to frying (Nacaroglu 2006; Wong et al. 2017). This pre‐treatment was applied to ensure consistent frying conditions; however, oil absorption of the fried potatoes was not evaluated, as the study focused on the deterioration behavior of the frying oils. Frying was performed in an industrial fryer (Remta R90, Turkey; 3 L capacity, 2.5 kW, 220 V, 50 Hz) containing 2 L of HO, PO, B‐1 (34% PO + 66% HO), B‐2 (66% PO + 34% HO), or S. The fryer was placed under a fume hood. Oil temperature was monitored using a thermocouple (Testo 175 T3, Germany). The fryer was set to 180°C. For each frying cycle, 250 ± 2 g of potato slices from the same batch were used. Each cycle consisted of frying the potatoes at 180°C for 6–7 min, followed by draining and removal of the fried product. Fresh potato slices were used for every frying cycle. Upon introduction of the potatoes, oil temperature dropped to ~135°C and then gradually increased, stabilizing around 160°C throughout the frying period. Between cycles, oil was allowed to cool to 100°C for approximately 45 min to simulate intermittent industrial usage, prevent thermal runaway, and allow sampling. After each cycle, 50 mL of oil was filtered (Whatman No. 1), transferred to amber glass bottles, flushed with nitrogen, and stored at +4°C until analysis. Four frying cycles were conducted per day using the same batch of oil. The frying experiments were distributed over three consecutive days to simulate intermittent frying conditions and to allow controlled cooling periods between frying sessions. This procedure was repeated for three consecutive days without replenishing the oil, resulting in a total of 12 frying cycles. The total number of frying cycles (12) was selected to allow sufficient monitoring of progressive oil deterioration during repeated frying while maintaining controlled laboratory conditions. By the end of the final cycle, the oil volume had decreased from 2.0 to approximately 1.1 L due to sampling (50 mL/cycle) and oil absorption by potatoes (≈10%). Fat content of fried potatoes was not analyzed as part of this study.

### 2.3 Free Fatty Acids

The FFA content was measured according to ISO 660:2020 (ISO 660 2020). Analyses were performed in triplicate. Results were expressed as % FFA, calculated as oleic acid for HO and HO‐rich blends and as palmitic acid for PO, PO‐rich blends, and S. Conversion factors were 0.503 (oleic acid equivalent) and 0.457 (palmitic acid equivalent).

### 2.4 Peroxide Value

The PV was determined according to the AOCS Official Method Cd 8–53 (AOCS 2017). The limit of detection and limit of quantification were 0.02 and 0.06 meq O_2_/kg oil, respectively. All analyses were performed in triplicate and reported as mean ± SD.

### 2.5 p‐Anisidine Value

The p‐AV analysis was carried out according to AOCS Official Method Cd 18–90 (AOCS 2024a). 0.5 g oil sample was weighed into a 25 mL graduated flask, completed with hexane up to the scale line. The absorbance of the test tube filled with hexane without sample was read at 350 nm (*A*_1_). 5 mL of the diluted hexane solution (0.5 g oil in 25 mL hexane) (m) was taken into the test tube. 1 mL p‐AV (0.25 g/100 mL glacial acetic acid) was added to the test tube. After 10 min the absorbance (*A*_2_) of the sample was read at 350 nm against the reference cell prepared with hexane and p‐AV without sample. The value of p‐AV was calculated according to the Equation (1).

(1) $$p-\mathrm{AV}=\frac{25\times \left[\left(1.2\times A_{2}\right)-A_{1}\right]}{m}$$

*m*: sample weight (g).

### 2.6 Total Oxidation Value

The TOTOX was calculated by adding 2 times PV with the p‐AV value (Equation 2) (Stauffer 1996).

(2) $$\text{TOTOX}=\left(2\times \mathrm{PV}\right)+p-\mathrm{AV}$$

### 2.7 Conjugated Diene and Triene

Conjugated dienes (K_232_) and trienes (K_270_) were determined according to AOCS Ch 5–91 (AOCS 2024b). A 0.25 g oil sample was dissolved in 25 mL isooctane and analyzed at 232 and 270 nm using a UV–Vis spectrophotometer (Agilent 8453, Agilent Technologies). The specific absorption values were determined using the following equation (Equation 3):

(3) $$E_{1\;\mathrm{cm}}^{\%1}=K_{\lambda }=\frac{A_{\lambda }}{c\times l}$$

where *K*_*λ*_, specific absorption value, *A*_*λ*_, absorbance value, c, concentration of solution (g/100 mL), l, quartz cuvette length (cm).

### 2.8 Fatty Acid Composition

The analysis was performed according to the fatty acid composition analysis protocol (AOCS 2009). A fat sample weighing 0.4 g was measured using a precision scale, and 4 mL of iso‐octane was subsequently added. The mixture was thoroughly mixed, and potassium hydroxide (KOH) prepared in 0.2 mL methanol was added. The resulting mixture was stirred again and left in the dark for 6 min. At the end of this period, 2–3 drops of methyl orange were added to the mixture, followed by the addition of 0.5 mL of 1 normal HCl solution. Finally, the entire mixture was left in the dark for 30 min, after which the clear liquid that formed at the top was collected into vials. Subsequently, 1 mL of this was injected into the injection port of a GC–MS device equipped with both an MS detector and an FID detector (Shimadzu GC/MS QP2010, Japan). The column specifications and operating conditions were as follows: a DB‐23 column (60 m × 0.25 mm, 0.25 μm); helium was utilized as the carrier gas with a total flow rate of 36.6 mL/min; the column flow rate was 0.66 mL/min; the linear velocity was 21.2 cm/s with a split ratio of 50. The initial temperature was set at 80°C and was increased by 10°C/min until it reached a final temperature of 220°C; both the injection and detection temperatures were maintained at 250°C. The entire analysis was completed in 34 min, with the ion source temperature set at 200°C. Fatty acid methyl esters were identified through chromatography using authentic standards (Sigma) and the NIST 05 MS Library Database. Quantification of the fatty acid methyl ester profiles was based on the relative peak areas, expressed as the relative percentage of each individual area in relation to the total area of compounds in the chromatogram. All measurements were performed in triplicate.

### 2.9 Color Analysis

Color parameters (L*, a*, b*) were measured at room temperature using a Minolta CR‐410 colormeter (Osaka, Japan). Color assessments of the samples were conducted following the procedure recommended by Öğütçü et al. (2008).

Each sample was measured three times and the average values were computed. The measurements yielded L* (lightness), a* indicating greenness (−) or redness (+), and b* representing blueness (−) or yellowness (+).

### 2.10 Total Polar Compounds

The TPC was measured using a Testo 270 device (Testo, Germany) after calibration. It should be noted that the abbreviation TPC may refer to two distinct parameters in oil analysis: total polar compounds and total phenolic content. However, these parameters represent different concepts. The proportion of total polar compounds is a key quality indicator that reflects the degree of oxidative degradation in frying oils and provides insight into their thermal stability, whereas total phenolic content is associated with the antioxidant capacity of the oil. In the present study, TPC specifically denotes total polar compounds. Prior to measurement, the device was calibrated using a specialized calibration oil. After each frying session, the oil samples were allowed to rest until air bubbles completely dissipated. Subsequently, the probe was immersed into three different points of the oil sample, and the mean value of the readings was recorded.

### 2.11 Viscosity

Viscosity measurements were conducted using a Haake Viscotester (İQ, Thermo Scientific, USA). For all measurements, the rotational speed and temperature were maintained at 95 rpm and 24°C, respectively. A total of 50 measurements were performed for each sample, and the average values were recorded. In this analysis, a CC25 DIN/Ti cylindrical probe was used with the device.

### 2.12 3‐MCPD and GE

3‐MCPD and GE levels were determined according to the standard method of DGF C VI 18 (10) (DGF 2011). Analyses were performed by applying the modifications detailed in the previous study (Gündüz et al. 2023).

### 2.13 Statistical Analysis

The results were analyzed using the SPSS software package (version 20.0 for Windows; SPSS Inc.). The variance analysis method was employed for evaluation, with the differences between means assessed using one‐way analysis of variance (ANOVA). Duncan's test was conducted to assess the significance of differences (*p* < 0.05) between applications.

## 3 Results and Discussion

### 3.1 Free Fatty Acids

**FIGURE 1:**
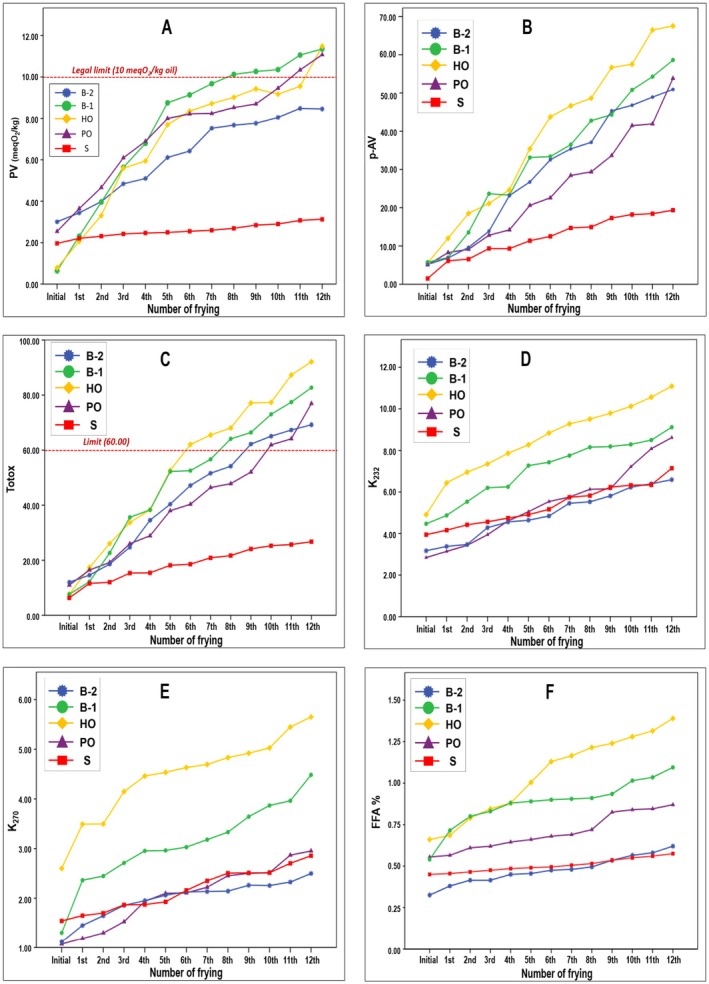
Changes in primary and secondary oxidation indices during 12 frying cycles. Variation in (A) peroxide value (PV), (B) *p*‐anisidine value (*p*‐AV), (C) total oxidation value (TOTOX), (D) conjugated diene content (K_232_), (E) conjugated triene content (K_270_), and (F) free fatty acids (FFA) of hazelnut oil (HO), palm olein (PO), blends B‐1 (34% PO + 66% HO) and B‐2 (66% PO + 34% HO), and shortening (S) during 12 deep‐frying cycles at 180°C. Values represent mean ± SD (*n* = 3). HO exhibited the highest oxidative degradation, while B‐2 and PO showed superior stability.

FFA measurement is widely used to monitor triglyceride hydrolysis during deep‐fat frying and to evaluate frying‐oil quality (Ceylan and Baştürk 2023). As shown in Figure 1F, initial FFA values did not differ significantly among the oils, indicating comparable starting quality. FFA levels increased in all samples with the number of frying cycles (*p* < 0.05). Initially, FFA levels ranged from 0.35% to 0.66% but increased to 0.63% to 1.39% by the end of the frying process (*p* < 0.05). At the beginning of the frying process, the FFA of PO was 0.61%, increasing gradually to 0.96% after twelve frying cycles. This steady rise reflects progressive hydrolysis of triglycerides due to the interaction of water released from the potato matrix with the oil at elevated temperatures. The increase in FFA values indicates that moisture accelerates ester bond cleavage, generating free fatty acids and partial acylglycerols during prolonged frying. During frying, FFA levels were higher in HO and B‐1 samples than in the other samples. In contrast, S and B‐2 treatments resulted in a lower increase in FFA levels. This behavior can be attributed to its higher content of saturated and monounsaturated fatty acids, which are less prone to hydrolytic breakdown than polyunsaturated fatty acids (Choe and Min 2007). However, HO, which is rich in unsaturated fatty acids, also showed a rapid increase in FFA. This is consistent with the observations of Cakmak‐Arslan and Gulsen (2024), who reported that oils with a higher degree of unsaturation tended to undergo hydrolysis more rapidly during continuous frying.

Although the FFA content of all samples remained below the practical rejection limit of ≈2% (Codex Alimentarius 1999), the progressive increase demonstrates ongoing hydrolytic deterioration. Elevated FFA levels not only contribute to the development of off‐flavors but also catalyze secondary oxidative reactions, thereby S the usable life of the oil (Silva et al. 2024).

### 3.2 Peroxide Value

The PV is a key indicator of primary oxidation products formed during the early stages of lipid oxidation. As presented in Figure 1A, PV increased in all oil samples with the number of frying cycles. The smallest increase was observed in S, where PV rose from 1.97 to 3.13 meq O_2_/kg after 12 cycles. In the other samples, PV increased more sharply. For most oils, except S, the rate of PV increase slowed after the 5th frying cycle. This behavior may be explained by the decomposition of hydroperoxides into secondary oxidation products such as aldehydes, ketones, and alcohols at frying temperatures (Bešter et al. 2008).

Previous studies also reported increasing PV during frying of PO (Che Man and Jaswir 2000) and HO (Karakaya and Simsek 2011). According to Turkish Food Codex (TGK 2012) and international regulations (Codex CXS 210; FSSAI; EU 2022/2104), PV in refined edible oils should not exceed 10 meq O_2_/kg. In the present study, this limit was exceeded in B‐1 at the 8th cycle (10.12 meq O_2_/kg), in PO at the 11th cycle (10.34 meq O_2_/kg), and in HO at the 12th cycle (11.49 meq O_2_/kg). However, a low PV alone does not necessarily indicate good quality, because hydroperoxides can decompose rapidly at frying temperatures, potentially underestimating oxidative deterioration (Baştürk 2019).

### 3.3 p‐Anisidine

The p‐AV metric assesses the advanced oxidative degradation of oils and fats by quantifying the secondary oxidation products of unsaturated fatty acids, primarily conjugated dienals and 2‐alkenals (Zhang et al. 2018). Figure 1B depicts the fluctuations in p‐AVs, which indicate secondary oxidation degradation products throughout the frying process. Throughout the frying process, an increase in p‐AV was observed in all oil samples. The most modest increase was noted in S, where the initial value of 1.48 rose to 19.35 by the conclusion of the 12th frying cycle. In contrast, the other oil samples exhibited initial p‐AV values ranging from 5.06 to 5.77, which escalated to values between 50.92 and 67.52 by the 12th frying cycle. The most pronounced increase was recorded for HO, followed by B‐1. The blend of HO and PO (B‐1 and B‐2) demonstrated greater efficacy than HO alone in mitigating or inhibiting the formation of p‐AV. Nonetheless, during frying, p‐AV was generated at reduced levels in both PO and S. Gündüz and Baştürk (2023) reported that the p‐AVs of HOs available in Turkish markets ranged from 2.2 to 5.2. The initial p‐AV of HO was slightly higher than these values, likely because of the storage conditions. The small differences in the initial PV and p‐AV values were attributed to the intrinsic oxidative history and refining processes (Cakmak‐Arslan and Gulsen 2024; Gündüz and Baştürk 2023).

### 3.4 Total Oxidation Value

The alterations in the TOTOX values of the oil samples throughout the frying process are depicted in the graph in Figure 1C. HO and B‐1 exhibited the most rapid oxidative deterioration. By the conclusion of the 12th frying cycle, the TOTOX values had reached approximately 92 and 82, respectively. B‐2 exhibited moderate oxidative deterioration with a TOTOX value of approximately 69. PO displayed a lower level of oxidative deterioration, with a TOTOX value of approximately 77. S resulted in the lowest TOTOX value of approximately 26. This phenomenon is directly associated with the fatty acid composition and degree of unsaturation of the oils. The oleic acid content in HO is substantial, ranging from 70% to 80%. Although oleic acid exhibits greater stability than linoleic acid, it is susceptible to accelerated oxidative degradation during extended frying at elevated temperatures. Consequently, although HO initially offers advantages, their oxidation rate significantly increases with continuous heating. PO, characterized by its high palmitic and oleic acid contents and low linoleic acid content, demonstrates superior oxidative stability compared to HO oils. The literature frequently recommends PO for frying stability. Being partially hydrogenated, S contains a high proportion of saturated fatty acids that are resistant to oxidation, thereby maintaining a low TOTOX level. However, the formation of trans fats has negative nutritional implications. The B‐1 and B‐2 blends exhibited moderate stability, contingent upon their respective mixing ratios. Specifically, B‐1, with a higher HO content, oxidized more rapidly, whereas B‐2, with a higher PO content, demonstrated greater resistance to oxidation. Although no regulatory limit has been established for TOTOX values, higher values generally indicate advanced oxidative deterioration because the index reflects both primary and secondary oxidation products (Choe and Min 2007; Stefani Juncos et al. 2024). According to the literature, after 12–20 frying cycles, the TOTOX values may range between 60 and 100 (Choe and Min 2007; Patil et al. 2023; Silva et al. 2024). Accordingly, the HO and B‐1 oils exceeded the limits after the 6th–7th frying. PO and B‐2, on the other hand, were relatively more resistant up to the 8th–9th frying. The higher palmitic + oleic acid content and lower polyunsaturated fatty acid (PUFA) fraction in PO and B‐2 provided greater oxidative stability by reducing hydroperoxide formation and polymerization (Akinoso et al. 2025; Patil et al. 2023; Romano et al. 2012). The S remained below the safety limits throughout the entire process. Although S is oxidatively the most stable method, the literature emphasizes that its use is currently restricted owing to health risks. The literature also reports that the oxidative behavior of blended oils depends on the saturated/unsaturated ratio in the composition (El‐gazzar et al. 2021; Kmiecik et al. 2022; Pizzimenti et al. 2023). In this study, the behavior of B‐2 being similar to that of PO confirms this result. In light of the literature, the use of PO or blended oils in frying appears to be more suitable in terms of both oxidative stability and health.

### 3.5 K_232_ and K_270_

The oxidation of hydroperoxides under unfavorable conditions can result in the displacement of double bonds, particularly in polyunsaturated fatty acids, thereby converting these fatty acids into a conjugated structure. This process may lead to the release of volatile compounds, such as aldehydes and ketones (conjugated trienes), into the environment owing to the breakdown of hydroperoxides (Ceylan and Basturk 2022). Figure 1D,E illustrate the variations in K_232_ and K_270_ levels throughout the frying cycle. Both parameters exhibited an upward trend with an increasing number of frying cycles (*p* < 0.05). The most significant increase in K_232_ levels was observed in HO, where the initial value of 4.91 rose to 11.09 by the conclusion of the 12th roasting cycle. Conversely, the smallest increase was recorded in B‐2, with an initial value of 3.17, which increased to 6.59 by the end of the 12th frying cycle. Notably, the increase was more pronounced in the HO and B‐1 samples, which can be attributed to their higher content of polyunsaturated fatty acids, specifically linoleic acid, compared to the other samples. In contrast, the degree of conjugation was lower in the S, B‐2, and PO oils. The K_232_ index is associated with the formation of conjugated dienes in polyunsaturated fatty acids, whereas K_270_ values indicate the presence of both primary and secondary oxidation products, including conjugated trienes and carbonyl compounds (Ceylan and Baştürk 2023).

### 3.6 Fatty Acid Composition

Table 1 summarizes the fatty‐acid profiles of the oils before and after 12 frying cycles. In HO, the predominant fatty acids were oleic, linoleic, palmitic, and stearic acids. Conversely, oleic, palmitic, linoleic, and stearic acids were predominant in PO, B‐1, B‐2, and S oils, respectively. The blended oils (B‐1 and B‐2) were formulated to closely approximate the fatty acid profile of S. Among all the oil samples, oleic acid was the most prevalent in HO (63.82%), followed by B‐1 (53.94%), B‐2 (47.59%), S (40.49%), and PO (40.14%). Following the twelfth frying cycle, the proportions of primary fatty acids exhibited minimal variation. Notable alterations included a reduction in linoleic acid in PO from 15.16% to 14.02% and a decrease in rumenic acid in HO from 0.43% to 0.07%. The preservation of the HO profile is consistent with the frying stability of high‐oleic oils (Porta and Aladedunye 2022). The other minor fluctuations were largely insignificant. The concentration of oleic acid remained constant across all oils. A significant decrease in linoleic acid was observed in the PO group, while other FAs remained stable. The moderate PUFA loss observed in palm fractions during long cycles was consistent with the literature (Patil et al. 2023). The B‐1 and B‐2 mixtures demonstrated intermediate stability between that of HO and PO. The oleic/linoleic ratio remained constant at around 3. The fluctuations in the oleic/linoleic ratios were minor and statistically insignificant, aligning with the anticipated “intermediate” behavior (Aşkın and Kaya 2020; Porta and Aladedunye 2022). The formation of oxidation and hydroperoxides during frying occurs more rapidly in polyunsaturated fatty acids. Consequently, the reductions in linoleic and linolenic acids are more pronounced than those in oleic acid. However, under practical conditions, changes in the percentage of fatty acids (FA) are often minor; quality loss is more commonly monitored through polar compounds and polymers, as the percentage of FA is a “slow” indicator. In the study, the reduction in linoleic acid in PO aligns with this general mechanism (Choe and Min 2007). The lack of significant differences in other oils has also been frequently reported in the literature. The presence of high oleic acid content enhances oxidative stability, as monounsaturated fatty acids (MUFAs) exhibit greater resistance to oxidation at elevated temperatures than polyunsaturated fatty acids (PUFAs) (Chen et al. 2023; Porta and Aladedunye 2022; Romano et al. 2021). Consequently, in HO and HO‐containing B‐1/B‐2 blends, the oleic content is anticipated to remain stable; findings corroborate this expectation.

**TABLE 1:** Fatty acid composition (%) of hazelnut oil (HO), palm olein (PO), blends (B‐1 and B‐2), and shortening (S) before frying and after the 12th frying cycle.

Samples→	HO		PO		B‐1		B‐2		S	
Fatty Acids↓	Before frying	After frying	Before frying	After frying	Before frying	After frying	Before frying	After frying	Before frying	After frying
Lauric	—	—	0.62 ± 0.11^a^	0.61 ± 0.07^a^	0.21 ± 0.06^a^	0.26 ± 0.05^a^	0.36 ± 0.08^a^	0.41 ± 0.08^a^	0.51 ± 0.04^a^	0.50 ± 0.07^a^
Myristic	0.07 ± 0.01^a^	0.07 ± 0.01^a^	2.08 ± 0.23^a^	2.11 ± 0.19^a^	0.70 ± 0.16^a^	0.80 ± 0.11^a^	1.24 ± 0.18^a^	1.41 ± 0.19^a^	1.87 ± 0.23^a^	1.96 ± 0.16^a^
Pentadecanoic	—	—	—	—	0.02 ± 0.03^a^	0.03 ± 0.04^a^	0.03 ± 0.04^a^	0.07 ± 0.01^a^	0.09 ± 0.01^a^	0.08 ± 0.01^a^
Palmitic	9.28 ± 0.34^a^	9.52 ± 0.49^a^	30.93 ± 0.31^a^	31.77 ± 0.62^a^	19.74 ± 0.01^a^	19.59 ± 0.06^a^	25.26 ± 0.42^a^	25.39 ± 0.34^a^	30.68 ± 0.69^a^	30.42 ± 0.27^a^
Palmitoleic	0.46 ± 0.06^a^	0.46 ± 0.10^a^	0.58 ± 0.16^a^	0.54 ± 0.04^a^	0.45 ± 0.09^a^	0.62 ± 0.12^a^	0.52 ± 0.07^a^	0.52 ± 0.11^a^	0.60 ± 0.06^a^	0.63 ± 0.02^a^
Margaric	—	0.06 ± 0.08	0.23 ± 0.04^a^	0.25 ± 0.06^a^	0.12 ± 0.04^a^	0.14 ± 0.02^a^	0.16 ± 0.03^a^	0.18 ± 0.03^a^	0.20 ± 0.04^a^	0.24 ± 0.06^a^
cis10Heptadecenoic	—	—	0.09 ± 0.03^a^	0.08 ± 0.01^a^	0.16 ± 0.05^a^	0.16 ± 0.03^a^	0.10 ± 0.01^a^	0.12 ± 0.02^a^	0.09 ± 0.04^a^	0.07 ± 0.00^a^
Stearic	4.54 ± 0.27^a^	4.91 ± 0.66^a^	7.95 ± 0.37^a^	7.95 ± 0.54^a^	5.31 ± 0.58^a^	5.92 ± 0.33^a^	6.40 ± 0.51^a^	6.94 ± 0.49^a^	7.61 ± 0.76^a^	7.89 ± 0.24^a^
Oleic	63.82 ± 1.30^a^	63.12 ± 2.38^a^	40.14 ± 1.00^a^	40.30 ± 0.90^a^	53.94 ± 1.80^a^	53.29 ± 0.92^a^	47.59 ± 1.34^a^	47.10 ± 1.07^a^	40.49 ± 0.68^a^	40.32 ± 0.47^a^
Linoleic	20.28 ± 0.24^a^	19.97 ± 0.29^a^	15.16 ± 0.11^a^	14.02 ± 0.34^b^	17.55 ± 0.23^a^	17.36 ± 0.08^a^	16.40 ± 0.35^a^	15.94 ± 0.12^a^	15.39 ± 0.52^a^	15.45 ± 0.28^a^
Linolenic	0.24 ± 0.03^a^	0.34 ± 0.05^a^	0.56 ± 0.06^a^	0.51 ± 0.19^a^	0.32 ± 0.08^a^	0.28 ± 0.02^a^	0.41 ± 0.05^a^	0.36 ± 0.04^a^	0.57 ± 0.06^a^	0.59 ± 0.13^a^
Arachidic	0.26 ± 0.04^a^	0.32 ± 0.06^a^	0.87 ± 0.11^a^	0.87 ± 0.11^a^	0.45 ± 0.08^a^	0.43 ± 0.06^a^	0.60 ± 0.10^a^	0.68 ± 0.10^a^	0.81 ± 0.16^a^	0.89 ± 0.06^a^
Rumenic	0.43 ± 0.06^a^	0.07 ± 0.10^b^	0.06 ± 0.01^a^	0.08 ± 0.01^a^	0.07 ± 0.01^a^	0.09 ± 0.01^a^	0.03 ± 0.04^a^	0.05 ± 0.06^a^	—	0.04 ± 0.05
cis11Eicosenoic	0.43 ± 0.06^a^	0.50 ± 0.13^a^	0.36 ± 0.05^a^	0.37 ± 0.06^a^	0.39 ± 0.08^a^	0.43 ± 0.05^a^	0.36 ± 0.06^a^	0.42 ± 0.08^a^	0.38 ± 0.08^a^	0.41 ± 0.06^a^
Behenic	—	—	—	0.09 ± 0.12	—	—	—	—	—	—
SFA	14.15	14.88	42.68	43.65	26.55	27.17	34.05	35.08	41.77	41.98
MUFA	64.71	64.08	41.08	41.21	54.78	54.34	48.47	48.04	41.47	41.36
PUFA	20.95	20.38	15.78	14.61	17.94	17.73	16.84	16.35	15.96	16.08
Oleic/Linoleic	3.15	3.16	2.65	2.87	3.07	3.07	2.90	2.95	2.63	2.61

*Note:* Values are presented as mean ± standard deviation (*n* = 3). “After frying” refers to the composition measured following the twelfth frying cycle. Different lowercase superscript letters within the same fatty acid row indicate significant differences between before‐ and after‐frying values for each oil type (*p* < 0.05).

Abbreviations: MUFA, monounsaturated fatty acids; PUFA, polyunsaturated fatty acids; SFA, saturated fatty acids.

### 3.7 Color Analysis

**FIGURE 2:**
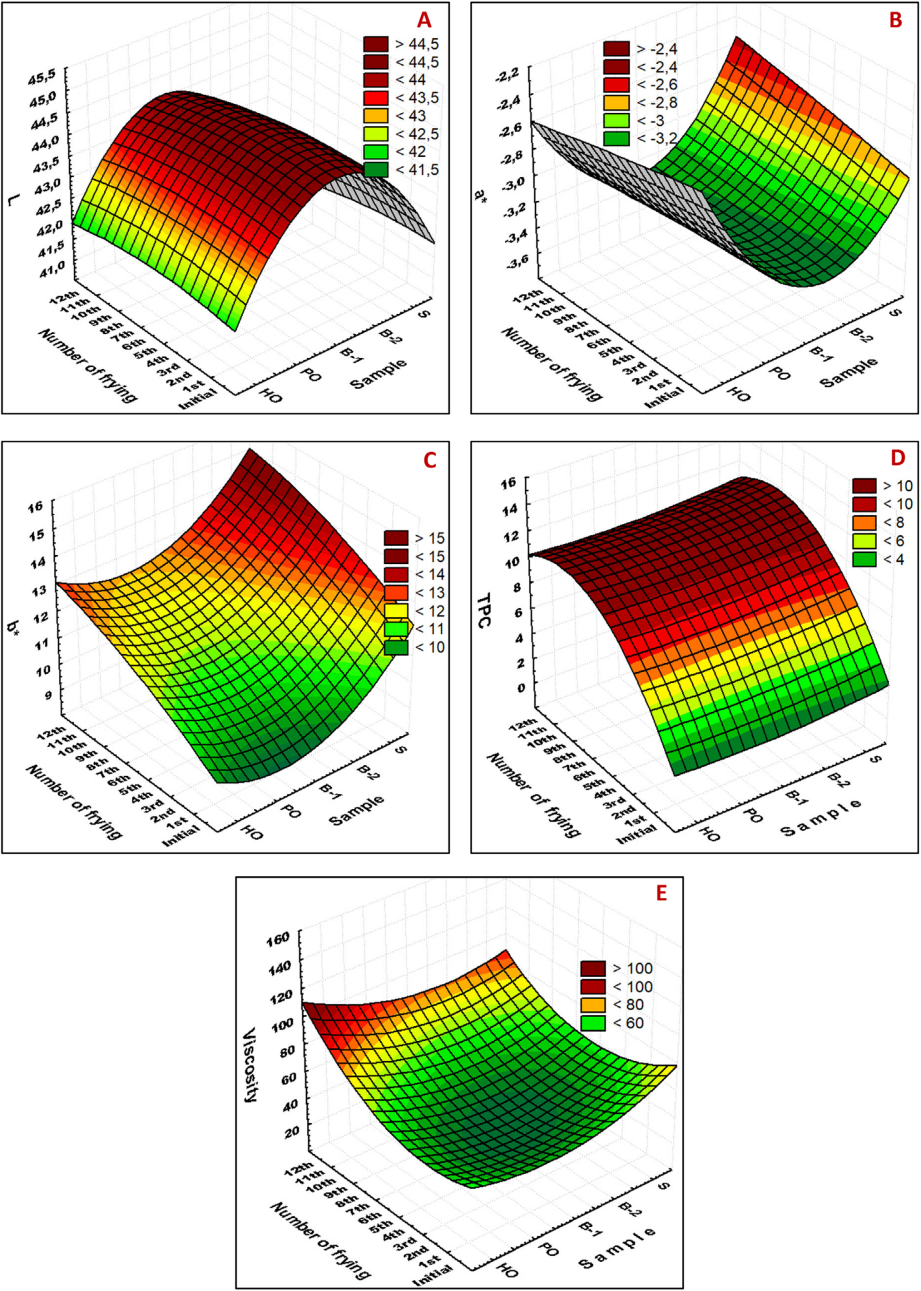
Changes in physical quality parameters during repeated frying. Variation in (A) color parameter L* (lightness), (B) a* (red–green axis), (C) b* (yellow–blue axis), (D) total polar compounds (TPC), and (E) viscosity of HO, PO, B‐1, B‐2, and S during 12 frying cycles. Increasing frying cycles caused progressive darkening (lower L*), shifts in a* and b* values, accumulation of TPC, and increased viscosity due to polymerization. All TPC values remained below the 25% rejection limit. Values are mean ± SD (*n* = 3).

The color changes of the oils during frying are shown in Figure 2. L* represents brightness, with a scale ranging from 0 (black) to 100 (white). The parameter a* indicates the red‐green spectrum, where +a denotes red and −a denotes green. Similarly, b* denotes the yellow‐blue spectrum, with +b* indicating yellow and −b* indicating blue. Several factors contribute to the color changes observed in oil samples, including the browning of melanoid pigments due to temperature, quantity of polyunsaturated fatty acids, darkening of color pigments in fried foods, conversion of fatty acids into a conjugated structure, temperature, number of frying cycles, and increase in polymer substances within the oil (Pedreschi et al. 2006). The redness value of the oil may be attributed to the oxidation of free fatty acids in the environment, while the yellowness, aldehydes, and peroxides, as well as the b* value, are associated with turbidity caused by the emulsified particles in the oil (Stier 2001). The darker color of the oil samples correlated with the number of frying cycles. The initial brightness values (L*) before frying ranged from 41.82 to 44.86 (Figure 2A). Generally, brightness decreased with an increase in the number of frying cycles (*p* < 0.05), with the exception of PO. This reduction in brightness may be attributed to the darkening of the oil as the number of frying cycles increased. The highest L* values were recorded for B‐1 and B‐2 before frying. The a* values of the pre‐frying samples ranged from −2.73 to −3.66 (Figure 2B). In HO, the a* value (ranging from −2.39 to −2.86) exhibited significant fluctuations (*p* < 0.05). The samples exhibiting the most pronounced green coloration prior to frying were PO (−3.66) and S (−3.41). The most notable alteration was observed in S, where the color transitioned from greenish (−3.41) to red (−2.65) as the frequency of frying in this oil increased (*p* < 0.05). The b* values, ranging from 8.87 to 14.12, were indicative of a yellow hue on the color scale (Figure 2C). Generally, these values exhibited an upward trend as the number of frying cycles increased. Specifically, after the 12th frying cycle, the b* values were observed to be within the range of 11.75 to 14.08.

### 3.8 Total Polar Compounds

Figure 2D illustrates the variation in the percentage of TPC during the frying process. The assessment of TPC in oils and fats is a widely recognized method due to its precision and reproducibility. It typically yields the most dependable results regarding the extent of degradation of frying oils and fats. Repeated frying procedures can elevate the TPC proportion in frying oil, which should not exceed 25% in frying oils (Başaran and Turk 2021). The maximum practically accepted limit for TPC was approximately 25%. This limit is a legal/operational threshold in EU countries, an official inspection threshold in Turkey, and a quantified form of the quality/deterioration criterion that technically underlies the Codex guidelines (Abrante‐Pascual et al. 2024; European Parliament/Joint Research Centre 2000; Firestone 2007). In certain countries, the TPC content in cooking oil is regulated to fall within the range of 24%–30% (Romano et al. 2012). Initially, the TPC ratios were zero, but they increased to a range of 10.50%–13.00% by the conclusion of the 12th frying cycle. A marked increase in TPC values was observed following the first frying, reaching levels between 7.67% and 8.83%. The TPC ratios continued to rise with subsequent frying cycles. These findings are consistent with data from previous studies (Casal et al. 2010; Karakaya and Simsek 2011). The most significant increase was noted in the PO group, followed closely by the S group. In the PO group, there was no significant difference in TPC values between the 1st and 6th frying cycles (7.67%–9.67%). In contrast, the increase in TPC was minimal for HO and B‐1 oils between the first and 12th frying cycles. Karakaya and Simsek (2011) reported that the TPC ratio increased significantly for various frying oils as the number of frying cycles increased. Importantly, none of the samples exceeded the TPC limits for frying, indicating that the oil samples were stable against thermal degradation.

### 3.9 Viscosity

The change in viscosity during the frying process is shown in Figure 2E. Viscosity characterizes the viscous properties of a liquid at a specified temperature under atmospheric pressure and standard gravity. Elevated temperatures in the frying pan result in increased polymer levels, leading to an increase in both oil viscosity and density (Peri and Saguy 2015). Because cooking oil functions as a heat‐transfer medium during frying, an increase in viscosity diminishes the heat‐transfer rate, thereby extending the required cooking time. Consequently, viscosity is a crucial parameter for assessing the quality of cooking oil. The viscosity of natural oils is significantly influenced by the degree of unsaturation and changes during frying and simple heating (Ceylan and Baştürk 2023; Romano et al. 2012). Generally, across all samples, the viscosity values increased with the number of frying cycles (*p* < 0.05). The initial viscosity values of PO and S oils were notably high compared to other samples (140.00 and 131.00 mPa s, respectively), which may be attributed to their solid state prior to frying. However, the viscosity of these oils decreased to 31.60 and 41.00 mPa·s following the first frying. The viscosity values, initially ranging from 21.30 to 43.96 mPa·s after the first frying, escalated to 69.89 to 96.81 mPa s by the conclusion of the twelfth frying cycle. The oil samples exhibiting the most significant increases in viscosity were B1 and HO. Comparable findings were reported by Tohma and Turan (2015).

### 3.10 3‐MCPD and GE

3‐MCPD and GE contents after repeated frying are summarized in Table 2. Levels of 3‐MCPD increased gradually in all oils over the frying period, with PO and S showing the highest accumulation of both 3‐MCPD and GE. HO exhibited the lowest increases, particularly for GE. The blends (B‐1 and B‐2) showed intermediate values: lower than PO and S but higher than HO. Before frying, GE concentrations ranged from 0.354 to 0.747 mg/kg. After 12 cycles, they increased significantly (*p* < 0.05) to 1.203–12.794 mg/kg, with the highest levels again in PO and S (12.687 and 12.794 mg/kg, respectively). These observations are consistent with previous findings that PO, its fractions, and S are high‐risk matrices for 3‐MCPD and GE (Goh et al. 2021; Gündüz et al. 2023; Mayayo et al. 2024). Although limited data exist for HO, available studies suggest that 3‐MCPD/GE formation in HO and other kernel oils is strongly affected by refining and diacylglycerol content, typically resulting in lower levels than in PO and S (Kantekin‐Erdogan et al. 2023). The present results support this pattern, with HO yielding the lowest contaminant levels. Several factors may explain the higher levels of 3‐MCPD and GE in S and PO: (i) PO, as a fruit oil, usually contains more moisture than seed oils, favoring hydrolysis and generation of mono‐ and diacylglycerols, which are precursors for 3‐MCPD and GE (Shahidi and Zhong 2005); (ii) organochlorine compounds in PO may act as chlorine sources (Nagy et al. 2011); (iii) palm trees can accumulate chloride ions from soil and water (Codex Alimentarius Commission 2018); and (iv) high‐temperature refining steps, especially deodorization, promote 3‐MCPD and GE formation (Franke et al. 2009; Weißhaar 2008).

**TABLE 2:** Levels of 3‐MCPD and GE (mg/kg) in different oil samples during repeated frying cycles.

	Number of frying	HO	PO	B‐1	B‐2	S
3‐MCPD	Initial	1.476 ± 0.037^aA^	2.243 ± 0.009^aD^	1.814 ± 0.014^aB^	2.122 ± 0.013^aC^	2.150 ± 0.004^aC^
	4th	2.876 ± 0.005^bA^	3.456 ± 0.005^bD^	3.007 ± 0.008^bB^	3.316 ± 0.012^bC^	3.465 ± 0.002^bD^
	8th	4.471 ± 0.003^cA^	6.837 ± 0.006^cD^	4.681 ± 0.005^cB^	5.195 ± 0.006^cC^	6.907 ± 0.005^cE^
	12th	5.096 ± 0.004^dA^	9.005 ± 0.002^dD^	7.392 ± 0.002^dB^	8.138 ± 0.002^dC^	9.116 ± 0.001^dE^
GE	Initial	0.747 ± 0.004^bD^	0.411 ± 0.001^aB^	0.546 ± 0.019^aC^	0.354 ± 0.004^aA^	0.401 ± 0.005^aB^
	4th	0.632 ± 0.002^aB^	0.702 ± 0.001^bD^	0.602 ± 0.004^bA^	0.663 ± 0.005^bC^	0.708 ± 0.004^bD^
	8th	0.906 ± 0.001^cA^	3.054 ± 0.001^cD^	1.098 ± 0.001^cB^	1.347 ± 0.000^cC^	3.250 ± 0.001^cE^
	12th	1.203 ± 0.040^dA^	12.687 ± 0.001^dD^	4.029 ± 0.001^dB^	6.321 ± 0.000^dC^	12.794 ± 0.001^dE^

*Note:* 3‐MCPD: 3‐monochloropropane‐1,2‐diol esters, GE: glycidyl esters, HO: hazelnut oil, PO: palm olein, B‐1: 34% PO + 66% HO, B‐2: 66% PO + 34% HO, S: shortening. Values are expressed as mean ± standard deviation (*n* = 3). Lowercase superscript letters within the same column indicate significant differences among frying cycles for each oil type (*p* < 0.05). Uppercase superscript letters within the same row indicate significant differences among oil types at a given frying cycle (*p* < 0.05). Statistical analyses were performed using one‐way ANOVA, followed by Duncan's multiple range test to determine significant pairwise differences.

Many studies report that increasing frying time or frequency is associated with higher GE levels. Under some conditions, GE continue to form while 3‐MCPD levels decrease due to hydrolysis or transfer into food (Wong et al. 2020). Other studies have shown diverse behaviors (initial increase then decrease, or continuous increase) depending on oil type, salt level, moisture, temperature, and oxidation degree (Kalkan et al. 2021; Xu et al. 2020; Yuan et al. 2022). Thus, no single pattern can be generalized for 3‐MCPD. In the present study, both 3‐MCPD and GE increased continuously in all oils, suggesting that formation predominated over degradation under the applied conditions.

## 4 Conclusion

This study comprehensively evaluated the oxidative stability, physicochemical changes, and contaminant formation behavior of HO, PO, their blends (B‐1 and B‐2), and S during 12 consecutive frying cycles. HO and HO‐rich blends exhibited the fastest deterioration, as reflected by higher increases in FFA, PV, p‐AV, TOTOX, K_232_, K_270_, viscosity, and color changes. PO and the PO‐rich blend B‐2 demonstrated superior oxidative stability, while S maintained the lowest oxidation levels but presented the highest formation of 3‐MCPD and GE, confirming its known risk profile. Across all oils, fatty‐acid compositions remained relatively stable during frying, with only modest reductions in PUFAs most notably in PO while oleic acid levels were largely preserved. TPC values remained below the regulatory rejection limit (25%) in all samples, indicating acceptable thermal stability under the applied conditions. Overall, PO and the B‐2 blend offered the best balance between oxidative stability and reduced contaminant formation, whereas HO and B‐1 were more prone to rapid oxidative degradation. The findings reinforce that oil blends containing higher proportions of PO are more suitable for extended frying applications, both in terms of quality preservation and food safety. These results contribute valuable insight for industry and consumers regarding the selection of frying oils, optimization of frying practices, and formulation of improved frying‐oil blends.

## Author Contributions

**Ayhan Baştürk:** writing – original draft, methodology, writing – review and editing, project administration, formal analysis, investigation. **M. Murat Ceylan:** investigation, writing – original draft, methodology, formal analysis, resources, data curation, writing – review and editing.

## Funding

This work was supported by Türkiye Bilimsel ve Teknolojik Araştırma Kurumu.

## Conflicts of Interest

The authors declare no conflicts of interest.

## Data Availability

The data that support the findings of this study are available from the corresponding author upon reasonable request.
